# Fragile X mental retardation protein promotes astrocytoma proliferation via the MEK/ERK signaling pathway

**DOI:** 10.18632/oncotarget.12215

**Published:** 2016-09-23

**Authors:** Zhou Xing, Minling Zeng, Huixian Hu, Hui Zhang, Zhuofang Hao, Yuesheng Long, Shengqiang Chen, Hang Su, Zhongmin Yuan, Meng Xu, Jingqi Chen

**Affiliations:** ^1^ Department of Oncology, the First Affiliated Hospital, Jinan University, Guangzhou 510632, People's Republic of China; ^2^ Translational Medicine Center, the Second Affiliated Hospital of Guangzhou Medical University, Guangzhou 510260, People's Republic of China; ^3^ Department of Medical Oncology, the Second Affiliated Hospital of Guangzhou Medical University, Guangzhou 510260, People's Republic of China; ^4^ Department of Neurosurgery, the Second Affiliated Hospital of Guangzhou Medical University, Guangzhou 510260, People's Republic of China; ^5^ Institute of Neuroscience; Key Laboratory of Neurogenetics and Channelopathies of Guangdong Province and Ministry of Education of China, Guangzhou Medical University, Guangzhou 510260, People's Republic of China; ^6^ Department of Pathology, the Second Affiliated Hospital of Guangzhou Medical University, Guangzhou 510260, People's Republic of China

**Keywords:** fragile X mental retardation protein, astrocytoma, MEK/ERK signaling pathway, proliferation, RNA interference

## Abstract

**Objective:**

To examine the association between fragile X mental retardation protein (FMRP) expression and astrocytoma characteristics.

**Methods:**

Pathologic grade and expressions of glial fibrillary acidic protein (GFAP), Ki67 (proliferation marker), and FMRP were determined in astrocytoma specimens from 74 patients. Kaplan-Meier survival analysis was undertaken. Pathologic grade and protein levels of FMRP were determined in 24 additional patients with astrocytoma and 6 controls (cerebral trauma). In cultured U251 and U87 cell lines, the effects of FMRP knock-down on cell proliferation, AKT/mTOR/GSK-3β and MEK/ERK signaling were studied. The effects of FMRP knock-down on the volumes and weights of U251 cell-derived orthotopic tumors in mice were investigated.

**Results:**

In patients, FMRP expression was increased in grade IV (5.1-fold, P<0.01) and grade III (3.2-fold, P<0.05) astrocytoma, compared with controls. FMRP and Ki67 expressions were positively correlated (R^2^=0.877, P<0.001). Up-regulation of FMRP was associated with poorer survival among patients with FMRP integrated optical density >30 (P<0.01). In astrocytoma cell lines, FMRP knock-down slowed proliferation (P<0.05), inhibited total MEK levels P<0.05, and reduced phosphorylation of MEK (Ser217/221) and ERK (Thr202/Tyr204) (P<0.05). In mice with orthotopic tumors, FMRP knock-down decreased FMRP and Ki67 expressions, and reduced tumor volume and weight (36.3% or 61.5% on day 15, both P<0.01). Also, phosphorylation of MEK (Ser217/221) and ERK (Thr202/Tyr204), and total MEK in xenografts were decreased in sh-FMRP xenografts compared with non-transfected ones (all P<0.05).

**Conclusion:**

Enhanced FMRP expression in astrocytoma may promote proliferation through activation of MEK/ERK signaling.

## INTRODUCTION

Gliomas are the most common type of primary brain tumors, accounting for approximately 80% of all malignant intracranial tumors [1]. Although they are a relatively rare form of tumor (an overall age-adjusted incidence rate of 4.67 to 5.73 per 100,000 persons), gliomas are associated with significant morbidity and mortality [1]. High-grade gliomas proliferate aggressively and are associated with poor prognosis [3].

Glioma proliferation is associated with the expression of functional proteins such as decoy receptor 3 (DcR3), a member of the tumor necrosis factor receptor super-family, and leucine-rich repeat containing G protein-coupled receptor 5 (LGR5) [4, 5]. In addition, constitutive activation of the mitogen-activated protein kinase/ERK kinase (MEK)/extracellular signal-regulated kinase (ERK) signaling pathway has been reported to promote the proliferation and migration of glioma cells [6].

Fragile X syndrome (FXS) is a monogenic disease that is associated with the expansion of the CGG trinucleotide repeat in the *Fragile X mental retardation-1* (*FMR1*) gene, resulting in a failure to express the Fragile X mental retardation protein (FMRP). FMRP is an RNA-binding protein that participates in the development of connections (synapses) between nerve cells [7–9]. FMRP enhances synaptic plasticity by transporting mRNA molecules within the neurons [10–12]. The failure to express FMRP results in a spectrum of intellectual disabilities (ranging from mild to severe), physical characteristics (such as elongated face or protruding ears), behavioral characteristics (such as stereotypic movements), and social anxiety [13]. Although the detailed mechanisms underlying the phenotype in FXS remain to be characterized, it has been reported that the absence of FMRP may lead to dysregulation of MEK/ERK and phosphoinositide 3-kinase (PI3K)/mammalian target of rapamycin (mTOR)/glycogen synthase kinase-3-beta (GSK-3β) signaling pathways [14–17].

FMRP expression is associated with cancer [18]. Overexpression of FMRP in breast cancer [19] and hepatocellular carcinoma [20, 21] is associated with a more aggressive metastatic phenotype. On the other hand, there is some evidence that patients with FXS may have an unusually low risk of brain tumor progression [22]. Because of this disparity between brain and non-brain tumors, this study aimed to examine the association between FMRP expression and astrocytoma proliferation.

## RESULTS

### FMRP expression in astrocytoma correlates with tumor proliferation

**Figure 1:**
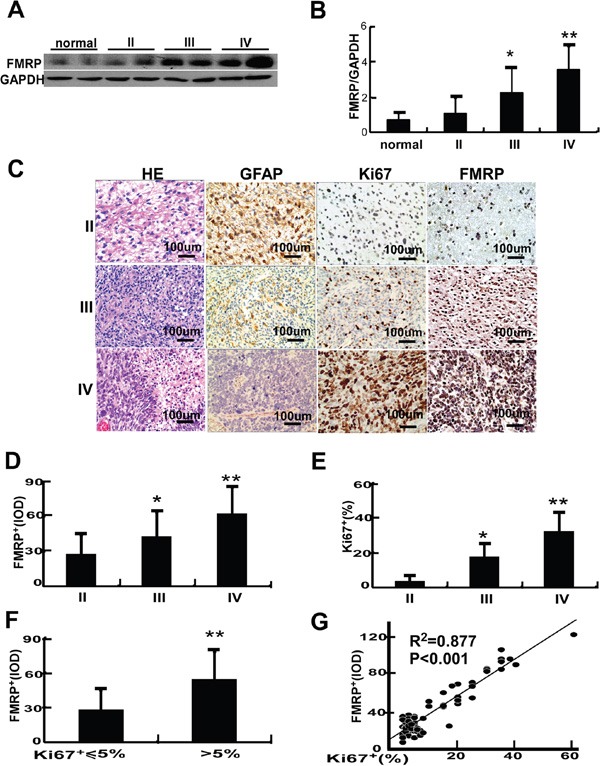
The expression of FMRP in astrocytoma tissues **A.** Representative image showing the detection of FMRP by western blot in brain tissue samples from patients with astrocytomas of varying pathologic grades (II, III or IV) or cerebral trauma (controls). GAPDH was used as a loading control. **B.** Mean data showing the expression of FMRP (relative to that of GAPDH) in brain tissue samples from patients with astrocytoma (grade II, III or IV) or cerebral trauma (controls). Bars correspond to the mean ± SD. * P<0.05, **P<0.01 vs. both the controls and grade II groups. **C.** Representative microscopy images showing brain tissue sections from patients with astrocytoma (grade II, III, or IV) stained with H&E or immunostained for GFAP, Ki67, or FMRP. **D.** FMRP^+^IOD values measured from immunostained brain tissue sections from patients with astrocytoma (grade II, III, or IV). Bars correspond to the mean ± SD. * P<0.05, ** P<0.01 vs. the grade II group. **E.** Percentage of Ki67^+^ cells measured from immunostained brain tissue sections from patients with astrocytoma (grade II, III, or IV). Bars correspond to the mean ± SD. * P<0.05, ** P<0.01 vs. the grade II group. **F.** FMRP^+^ IOD data grouped according to the percentage of Ki67^+^ cells (≤5% or >5%). Values are presented as the mean ± SD. ** P<0.01 vs. Ki67^+^ ≤5% cells. **G.** Correlation between FMRP^+^ IOD and percentage of Ki67^+^ cells in astrocytoma tissue.

Western blot was used to detect FMRP protein expression in tissue samples from 24 patients with astrocytoma and 6 patients with cerebral trauma (as a control). Compared with controls, the FMRP protein levels were increased by 5.1-fold in grade IV astrocytoma (P<0.01) and by 3.2-fold in grade III tumors (P<0.05; Figure 1A and 1B).

Immunohistochemistry (IHC) in samples from 74 patients revealed that GFAP expression (a marker of glioma cell differentiation) decreased, while the levels of FMRP and Ki67 increased with grade (Figure 1C). The integrated optical density (IOD) of FMRP^+^ cells and the proportion of Ki67^+^ cells were, respectively, 27.9 and 3.8% for grade II tumors, 43.1 and 17.8% for grade III tumors (both P<0.05 vs. grade II), and 62.8 and 32.2% for grade IV tumors (both P<0.01 vs. grade II; Figure 1D and 1E). The IOD of FMRP^+^ cells was 28.2 in samples with Ki67^+^ ≤5%, but 54.4 in samples with Ki67^+^ >5% (P<0.01; Figure 1F). FMRP expression correlated positively with Ki67 expression (R^2^=0.877, P<0.001; Figure 1G).

**Figure 2:**
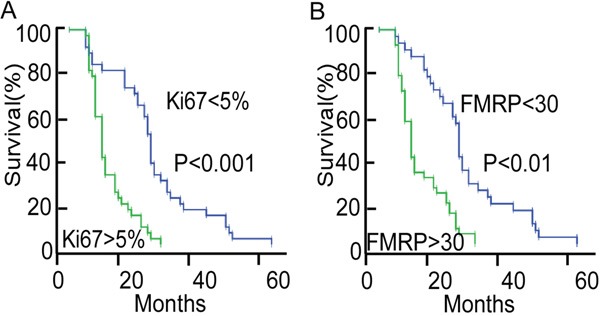
Analyses of overall survival in patients with astrocytoma according to the Ki67 or FMRP status **A.** Kaplan-Meier survival curve for patients with astrocytoma, grouped according to the percentage of Ki67^+^ tumor cells (≤5%, n=36; or >5%, n=38). The median follow-up was 16.3 months. **B.** Kaplan-Meier survival curve for patients with astrocytoma, grouped according to the IOD of FMRP^+^ tumor cells (≤30, n=32; or >30, n=42). The median follow-up was 16.3 months.

Kaplan-Meier analysis (median follow-up, 16.3 months; range, 5-62 months; Figure 2) revealed better survival in patients with a low percentage of Ki67^+^ tumor cells (P<0.001, ≤5% vs. >5%) or low IOD of FMRP^+^ cells (P<0.01, ≤30 vs. >30). Furthermore, higher FMRP^+^ IOD was associated with bigger tumor size (P<0.05, ≤30 cm^3^ vs. >30 cm^3^), higher grade tumors (P<0.05, grade II vs. grade III/IV), and higher expression Ki67 (P<0.05, ≤5% vs. >5%), but not with sex or age (Table 1).

**Table 1:** Correlation of the average integrated density of FMRP^+^ cells with clinicopathological status in 74 patients with astrocytoma

FMRP^+^ IOD	≤ 30 (n=32)	> 30 (n=42)	P value
Sex			
Male	15	21	0.790
Female	17	21	
Age			
≤37	19	20	0.316
>37	13	22	
Tumor size (cm^3^)^a^			
≤30	20	16	0.037
>30	12	26	
Pathological grade^b^			
II	22	19	0.044
III-IV	10	23	
Ki-67^c^			
≤5%	22	14	0.003
>5%	10	28	

a Tumor size identified by magnetic resonance imaging before surgery and calculated with formula V=L×W×H/2 cm^3^ (V, volume; L, length; W, width; H, height).

b Grading of the 74 cases of astrocytoma.

c Ki67 expression in astrocytoma tissue determined using immunohistochemistry.

### FMRP may promote the proliferation of astrocytoma cells via MEK/ERK signaling

**Figure 3:**
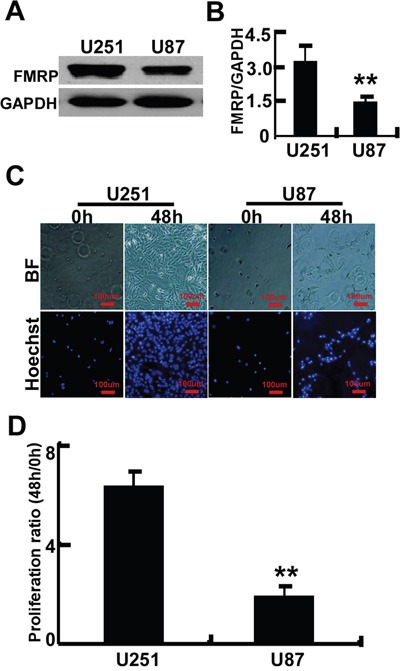
FMRP expression and proliferation of two astrocytoma cell lines **A.** Representative image showing the detection of FMRP by western blot in U251 and U87 cell lines. GAPDH was used as a loading control. **B.** Mean data showing the expression of FMRP (relative to that of GAPDH) in U251 and U87 cells. Bars correspond to the mean ± SD of triplicate experiments. **P<0.01. **C.** Representative microscopy images showing U251 and U87 cells before and after culture for 48 h. BF, bright-field; Hoechst, nuclei visualized with Hoechst stain. **D.** Proliferation ratios (ratio of cell count at 48 h to that at 0 h) for U251 and U87 cells, determined from experiments such as those in panel (C). Values are presented as the mean ± SD of triplicate experiments. ** P<0.01.

The relationship between FMRP expression and tumor proliferation was further investigated in astrocytoma cell lines. Western blot revealed that FMRP expression was higher in U251 cells than in U87 cells (P<0.01, Figures 3A and 3B). Moreover, when the same number of cells (5×10^4^/well) were cultured for 48 hr, the proliferation of U251 cells was 3.3-fold higher than that of U87 cells (P <0.01; Figures 3C and 3D).

**Figure 4:**
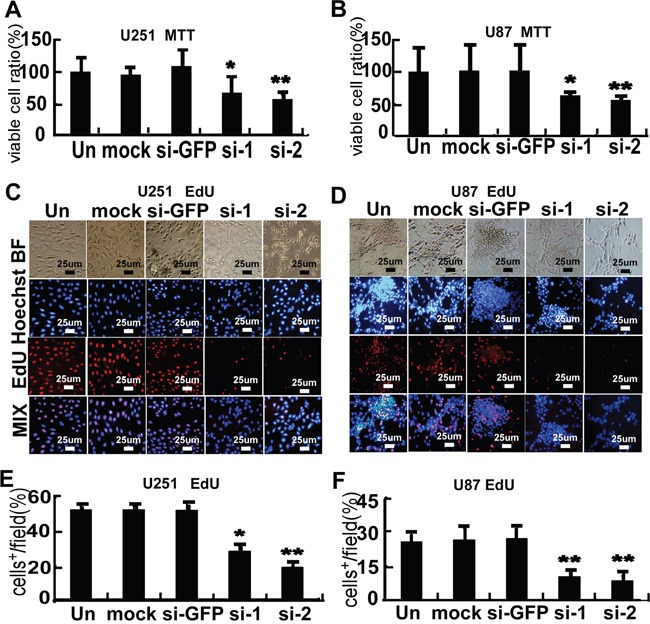
FMRP promotes proliferation of astrocytoma cell lines **A.** MTT cell proliferation assay for U251 cells that were not transfected (Un), mock transfected (mock), transfected with either of two FMRP-siRNAs (si-1, si-2), or transfected with GFP-siRNA (si-GFP). **B.** Corresponding data for U87 cells. Values are presented as the mean ± SD of triplicate experiments. * P<0.05, ** P<0.01 vs. non-transfected cells. **C.** Representative microscopy images showing U251 cells that were non-transfected (Un), mock transfected (mock), transfected with either of two FMRP-siRNAs (si-1, si-2), or transfected with GFP-siRNA (si-GFP). BF, bright-field; Hoechst, nuclei visualized with Hoechst stain; EdU, cells stained with EdU; MIX, superimposed images for Hoechst and EdU. **D.** Corresponding data for U87 cells. **E.** Percentage of EdU-incorporating U251 cells that were not transfected (Un), mock transfected (mock), transfected with either of two FMRP-siRNAs (si-1, si-2), or transfected with GFP-siRNA (si-GFP). **F.** Corresponding data for U87 cells. Values are presented as the mean ± SD of triplicate experiments. * P<0.05, ** P<0.01 vs. non-transfected cells.

Transfection of cells with two different FMRP-siRNAs (separately) resulted in decreases in proliferation (measured by the MTT assay) of 33.3% and 43.0% in U251 cells (P<0.05 and P<0.01 vs. untreated cells), and 37.1% and 44.1% in U87 cells (P<0.05 and P<0.01 vs. untreated cells), respectively (Figure 4A and 4B). There were no significant effects of mock transfection or transfection of si-GFP in these cells (Figure 4A and 4B). Similar results were obtained using EdU incorporation to assess cell proliferation (Figures 4C-4F). Transfection with FMRP-siRNAs reduced the number of EdU-positive cells by 46.0% and 63.9% in U251 cells (P<0.05 and P<0.01 vs. untreated cells, Figure 4E), and 59.7% and 66.2% in U87 cells (both P<0.01 vs. untreated cells, Figure 4F), respectively.

**Figure 5:**
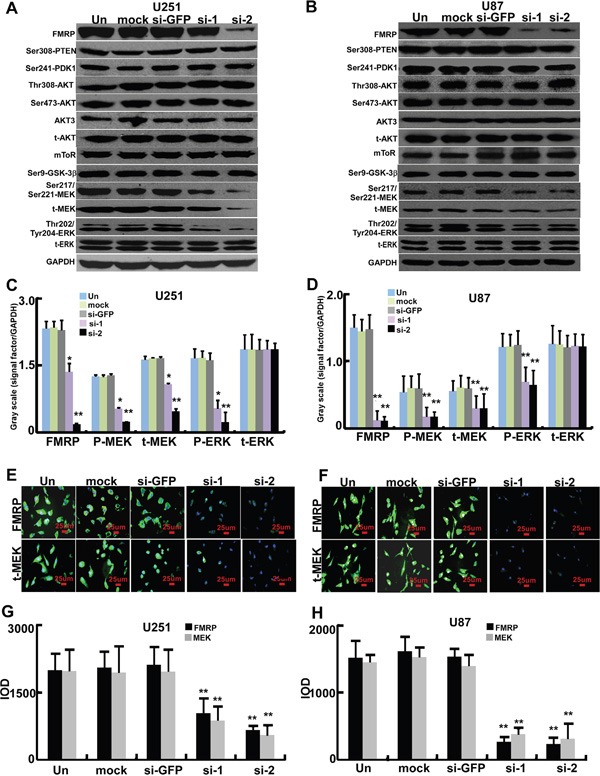
FMRP induces MEK/ERK activation in astrocytoma cells **A.** Representative western blots from U251 cells that were not transfected (Un), mock transfected (mock), transfected with either of two FMRP-siRNAs (si-1, si-2), or transfected with GFP-siRNA (si-GFP). Phosphorylated and total protein expressions of FMRP, PTEN, PDK1, AKT, mTOR, GSK-3β, MEK, and ERK are shown. GAPDH was used as a loading control. **B.** Corresponding data for U87 cells. **C.** Mean data showing the expressions of FMRP, total MEK (t-MEK), phosphorylated MEK (P-MEK), total ERK (t-ERK) and phosphorylated ERK (P-ERK) (relative to that of GAPDH) in U251 cells that were not transfected (Un), mock transfected (mock), transfected with either of two FMRP-siRNAs (si-1, si-2), or transfected with GFP-siRNA (si-GFP). **D.** Corresponding data for U87 cells. Values are presented as the mean ± SD of triplicate experiments. * P <0.05, **P<0.01 vs. non-transfected cells. **E.** Representative images showing immunofluorescence for FMRP and total MEK (t-MEK) in U251 cells that were not transfected (Un), mock transfected (mock), transfected with either of two FMRP-siRNAs (si-1, si-2), or transfected with GFP-siRNA (si-GFP). **F.** Corresponding data for U87 cells. **G.** Mean data determined from the immunostaining experiments, showing the levels of FMRP and MEK inU251 cells that were not transfected (Un), mock transfected (mock), transfected with either of two FMRP-siRNAs (si-1, si-2), or transfected with GFP-siRNA (si-GFP). **H.** Corresponding data for U87 cells. Values are presented as the mean ± SD of triplicate experiments. **P<0.01 vs. non-transfected cells.

Further experiments were performed to explore the effects of decreased FMRP expression on signaling pathways. Transfection of either of the two FMRP-siRNAs into U251 or U87 cells did not alter the phosphorylation of PTEN at Ser308, PDK1 at Ser241, AKT at Thr308 or Ser473, or GSK-3β at Ser9, suggesting no effect of FMRP-siRNAs on AKT/mTOR/GSK-3β signaling (Figure 5A and 5B). AKT3 mRNA is transported by FMRP from the nucleus to the cytoplasm [23], but the total protein level of AKT3 remained unchanged in U251 and U87 cells after FMRP knockdown (Figure 5A and 5B). Interestingly, both FMRP-siRNAs inhibited total MEK levels and reduced the phosphorylation of MEK at Ser217/221 and ERK at Thr202/Tyr204 (P<0.05 and P<0.01). GFP-siRNA and mock transfection were without effect (Figures 5A-5D). Immunostaining revealed that MEK expression in U251 and U87 cells was reduced by both FMRP-siRNAs (P<0.01), with no effect of GFP-siRNA or mock transfection (Figure 5E–5H).

### FMRP promotes the growth of astrocytoma *in vivo*

**Figure 6:**
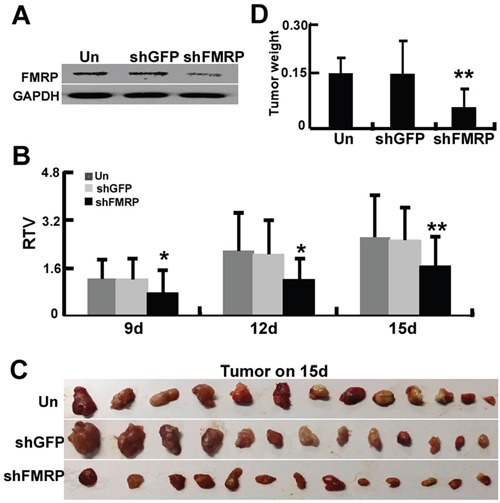
FMRP promotes growth of astrocytoma xenografts *in vivo* **A.** Representative western blots showing FMRP expression in U251 cells that were either infected or not with lentivirus vector carrying GFP-shRNA or FMRP-shRNA. GAPDH was used as a loading control. **B.** U251 cells were either infected or uninfected with lentivirus vector carrying GFP-shRNA or FMRP-shRNA, and inoculated into the pads of nude mice to develop orthotopic tumors. Relative tumor volumes (RTV) were measured at the indicated times. Bars correspond to the mean ± SD (*n*=13 for each group). * P <0.05, ** P <0.01 vs. non-transfected or GFP-shRNA transfected group. **C.** Examples of orthotopic tumors obtained from the mice on day 15. **D.** Orthotopic tumor weights on day 15. Values are presented as the mean ± SD (*n*=13 for each group). ** P <0.01 vs. non-transfected or GFP-shRNA transfected group.

To examine the effect of FMRP on astrocytoma growth *in vivo*, U251 cells were either infected or uninfected with lentivirus vector carrying GFP-shRNA or FMRP-shRNA, and inoculated into nude mice to develop orthotopic tumors. Western blot experiments confirmed that lenti-FMRP-shRNA, but not lenti-GFP-shRNA, inhibited FMRP expression in U251 cells (Figure 6A). The Relative tumor volumes of U251 cell xenografts infected with lenti-FMRP-shRNA were reduced by 37.2% on day 9 (P<0.05), 44.2% on day 12 (P<0.05), and 36.3% on day 15 (P<0.01), as compared with uninfected xenografts (Figures 6B and 6C). Furthermore, the mean weight of the tumors derived from cells infected with lenti-FMRP-shRNA was reduced by 61.5% on day 15 (P<0.01; Figure 6D).

**Figure 7:**
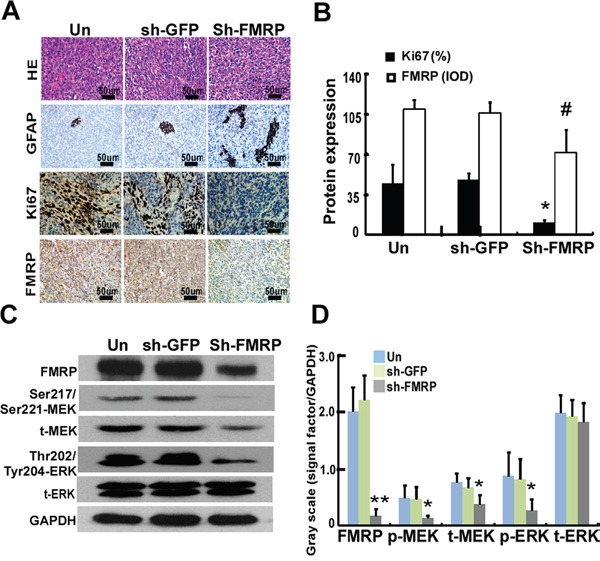
FMRP expression correlates to Ki67 expression and MEK/ERK signaling pathway *in vivo* **A.** Representative microscopy images showing sections of orthotopic tumors from mice, stained with H&E or immunostained for GFAP, Ki67, or FMRP. The xenograft tumors were derived from U251 cells either infected or not with lentivirus vector carrying GFP-shRNA or FMRP-shRNA. **B.** IOD analysis for the expressions of Ki67 and FMRP in xenograft tumors, derived from U251 cells that were either transfected or not with GFP-shRNA or FMRP-shRNA. Values are presented as the mean ± SD (*n*=13 for each group). *^, #^ P<0.05 vs. non-transfected or GFP-shRNA transfected mice. **C.** Western blot analysis of FMRP, Ser217/Ser221-MEK, Thr202/Tyr204-ERK, total MEK, and total ERK. **D.** Quantification of C. Values are presented as the mean ± SD (n=5 for each group). *P<0.05, **P<0.01 vs. non-transfected (Un) or GFP-shRNA transfected (sh-GFP) group.

IHC experiments on orthotopic mouse tumors revealed that xenografts from U251 cells infected with lenti-FMRP-shRNA exhibited decreased expressions of FMRP and Ki67, and increased expression of GFAP (Figure 7A). Overall, FMRP knockdown was associated with a 77.1% decrease in Ki67 expression (P<0.05) and a 34.7%. reduction in FMRP expression (P<0.05; Figure 7B). After FMRP silencing, the levels of Ser217/Ser221-MEK, Thr202/Tyr204-ERK, and total MEK in xenografts were decreased in sh-FMRP xenografts compared with non-transfected ones (all P<0.05). The sh-FMRP has no effect on total ERK levels.

## DISCUSSION

The main findings of the present study were that in patients with astrocytoma, FMRP expression was associated with higher pathologic grade, faster proliferation (assessed from Ki67 expression), larger tumor size and shorter survival. Furthermore, knockdown of FMRP reduced cell proliferation and inhibited MEK/ERK signaling in astrocytoma cell lines, and decreased the growth of xenograft tumors in mice. Taken together, these data suggest that FMRP may promote the proliferation of astrocytoma cells through the activation of MEK/ERK signaling.

The expansion of the CGG trinucleotide repeats in the 5' untranslated region (UTR) of *FMR1* results in failure of FMRP expression [24]. In unaffected individuals, the 5' UTR of *FMR1* contains 5-44 repeats of the CGG codon, whereas individuals with FXS have a mutated *FMR1* allele with over 200 repeats of the CGG codon [25]. The present study showed that the expression of FMRP in astrocytoma does not correlate with the number of CGG trinucleotide repeats in the 5′ UTR of the *FMR1* gene (data not shown), but does increase with increasing tumor grade and Ki67 expression. Moreover, the data indicated that FMRP expression correlates with the astrocytoma proliferation. To our knowledge, this is the first study to reveal the relationship between FMRP expression and the biological characteristics of astrocytoma.

FMRP is an RNA-binding protein that targets distinct mRNA sequence elements to increase the expression of proteins in neurons [23]. Blocking the expression of FMRP impairs synaptic delivery and induces mild or profound behavioral disorders and learning defects [32], leading to FXS, mental retardation, premature ovarian failure, autism, Parkinson's disease, developmental delays, and cognitive deficits [33–35]. FMRP is overexpressed in hepatocellular carcinoma cells [20], and the expression of FMRP correlates with the prognosis and lung metastasis of invasive breast cancer [19], suggesting a role of FMRP in cancer. In the present study, the expression of FMRP was elevated in high-grade astrocytoma, and correlated with Ki67 expression and poor prognosis. Despite advances in surgery, radiotherapy, and chemotherapy, the prognosis of patients with glioma remains poor [26]. The molecular characteristics of individual tumors, such as the level of activated PTEN, PI3K, AKT, mTOR, and ERK, are important prognostic factors for patients with glioma [27–31]. The present study showed that FMRP promotes the proliferation of astrocytoma both *in vitro* and *in vivo*, and that its expression levels correlate with poor prognosis.

FMRP has a crucial function in neuronal signal transduction as it mediate the expressions of many signaling factors that affect the functional status of molecular signaling networks [36–38]. The action of FMRP on several molecules involved in neurotransmitter-induced signaling pathways have been studied, including GSK-3β [39, 40], matrix metallopeptidase-9 (MMP-9) [41], PAK and regulator of G-protein signaling-4 (RGS4) [42]. ERK1/2 and PI3K/mTOR are two major pathways implicated in dysregulated neurotransmitter-dependent signaling in FXS [16, 43]. ERK1/2 activation by metabotropic glutamate receptors-1/5 (mGlu1/5) is deficient in FXS [14], and the PI3K/mTOR signaling response to mGlu1/5 stimulation is lost [43]. In the present study, knockdown of FMRP expression did not inhibit the activation of the PI3K/mTOR/GSK-3β signaling pathway. Instead, decreased FMRP expression resulted in an inhibition of MEK expression. These findings are consistent with the proposal that the enhanced expression of FMRP in astrocytoma cells may promote tumor proliferation through activation of the MEK/ERK signaling pathway.

In summary, our study shows that increased expression of FMRP, an important RNA binding protein, is associated with increased astrocytoma proliferation. Further studies are needed to determine the molecular mechanisms linking the elevation of FMRP expression to increased Ki67 expression and enhanced astrocytoma proliferation.

## MATERIALS AND METHODS

### Patients and tissue samples

We retrospectively reviewed the electronic medical records and radiology information systems of the No. 2 Affiliated Hospital, Guangzhou University, China to identify patients diagnosed with an astrocytoma between January 2004 and October 2014. The inclusion criteria were: 1) histopathologic diagnosis of astrocytoma without oligodendroglial components, according to World Health Organization (WHO) criteria [2]; and 2) 3-T magnetic resonance (MR) imaging with dynamic susceptibility contrast perfusion weighted imaging (DSC-PWI) was carried out prior to surgery or chemoradiotherapy. Of the 77 patients screened for inclusion, three were excluded: 1) the paraffin tissue block was too small to enable further study (*n*=2); or 2) the MR imaging quality was inadequate due to substantial motion artifact (*n*=1).

A total of 74 patients (median age, 37 years; age range, 2–71 years) were included in the study, and serial sections of their astrocytoma were obtained. Among the 74 enrolled patients, 41 had WHO grade II astrocytoma, and 33 had WHO grade III or IV astrocytoma. Grade III astrocytomas and grade IV astrocytomas (glioblastomas) were classified as high-grade gliomas, while grade II astrocytomas were categorized as low-grade gliomas. The pathologic diagnosis with glial fibrillary acidic protein (GFAP), Ki67, and FMRP status were verified by two different pathologists. Tumor length, width and height was measured on MRI films and tumor size was calculated (tumor size=length×width×height/2). Patients with high-grade gliomas received radiotherapy after surgery, and subsequently underwent chemotherapy with temozolomide (150 mg/m^2^) [44]. The median follow-up period was 16.3 months (range, 5-62 months).

Glioma tissue samples were obtained from 24 patients, for use in an additional series of experiments. These patients had undergone surgical resection of their glioma in our institution between March 2013 and March 2014, and met the inclusion criteria described above. Additional brain tissue samples, which were used as controls, were obtained from six patients with cerebral trauma.

The study was approved by the Internal Review and Ethics Boards of the Second Affiliated Hospital of Guangzhou University, China. All patients or legal guardians provided informed consent for inclusion in the study.

### Cell culture, treatment and transfection

U251 and U87 astrocytoma cells were obtained from the American Type Culture Collection (ATCC, MA, USA) and cultured in Dulbecco's modified Eagle's medium (Sigma, USA) with 10% fetal bovine serum (FBS, Sigma, USA), 100 U penicillin, 1000 U streptomycin, 2 mM L-glutamine, 1mM Na-pyruvate, and 0.1 mM non-essential amino acids. Cells were plated at a density of 15,000 cells/cm^2^. The U251 and U87 glioma cells were transfected with FMRP-siRNA in serum-free OPTI-MEMI (Invitrogen, CA, USA) using Lipofectmine 2000 reagent (Invitrogen), according to the manufacturer's instructions. All siRNAs were synthesized using 2′-O-ACE phosphoramiditis (RiboBio, Guangzhou, China). The sense and anti-sense strands of the siRNAs are detailed in Supplemental Table S1.

### Immunohistochemistry

IHC was performed using standard labeled streptavidin-biotin (LSAB) protocols (Dako, Carpinteria, CA) on paraffin sections of glioma tissue. The primary antibodies were rabbit polyclonal antibodies against FMRP (1:200, Abcam, UK), GFAP (1:50, Abcam, UK), and Ki67 (1:50, Abcam, UK). Isotype-matched antibodies were applied as negative controls. The staining of cells with FMRP or Ki67 was quantified as the IOD or percentage(%) per field of view, using Image-ProPlus 6.0 (Media Cybernetics, Inc., MD, USA). At least 20 view-fields per section were evaluated at 400× magnification.

### Western blot

Protein extracts were resolved by 8% sodium dodecyl sulfate-polyacrylamide gel electrophoresis (SDS-PAGE), transferred to polyvinylidene difluoride (PVDF) membranes, and probed with antibodies against human FMRP, phosphatase and tensin homolog (PTEN), phosphoinositide-dependent protein kinase-1 (PDK1), AKT, mTOR, GSK-3β, MEK, ERK, the corresponding phosphorylated proteins (Cell Signaling Technology (CST), MA, USA), and glyceraldehyde 3-phosphate dehydrogenase (GAPDH, CST). Peroxidase-conjugated anti-mouse or rabbit IgG (CST) was used as the secondary antibody. The antigen-antibody reaction was visualized with an enhanced chemiluminescence (ECL) assay (Thermo Fisher Scientific, MA, USA). The gray values of protein were analyzed with ImageJA software (National Institutes of Health, USA).

### EdU incorporation assay

After knock-down of FMRP for 48 hr, U251 cells (5×10^4^ cells/well) and U87 cells (5×10^4^ cells/well) were seeded in 24-well plates. To evaluate the proliferation of the glioma cells, 50 μM 5-ethynyl-2′-deoxyuridine (EdU; Ribobio, China) was added to the medium for 2 h. To determine the incorporation of EdU, the cells were fixed with 4% paraformaldehyde for 30 min at room temperature, and immunostained (red) using a standard protocol; in addition, Hoechst stain (blue) was used to visualize the nucleus. EdU incorporation was calculated per field of view, with at least 20 view-fields per section evaluated by IOD at 400× magnification.

### MTT assay

The proliferation of glioma cells was determined using the MTT (3-(4,5-dimethylthiazol-2-yl)-2,5-diphenyltetrazolium bromide) assay. In brief, U251 cells (5×10^3^ cells/well) and U87 cells (5×10^3^ cells/well) were transfected with FMRP-siRNA and incubated for 48 h in a 96-well plate. MTT solution (5 mg/mL, 20 μL) was added and the plate incubated for 4 h. Dimethyl sulfoxide (DMSO: 150 μL) was used to dissolve the formazan crystals. The absorbance was measured at 490 nm with a microplate reader (Asys UVM340; Eugendorf, Austria).

### Immunofluorescence

For immunofluorescence experiments, the cells were incubated with primary antibody against FMRP or MEK (CST), followed by incubation with Alexa488-conjugated goat antibody against rabbit IgG (Life Technologies, CA, USA). For immunofluorescence microscopy, the cells were plated on cover-slips, counterstained with 4',6-diamidino-2-phenylindole (DAPI), and imaged using an immunofluorescence microscope (Olympus, Japan). The fluorescence signals of cells with FMRP or MEK was quantified as the IOD per field of view, using Image-ProPlus 6.0 (Media Cybernetics). At least 20 view-fields per section were evaluated at 400× magnification.

### Tumor xenografts

Male nude mice were bred and maintained under defined conditions at the Animal Experiment Center of Guangzhou Medical University. All procedures were approved by the Animal Care and Use Committee of the No. 2 Affiliated Hospital, Guangzhou Medical University, and conformed to the legal mandates and national guidelines for the care and maintenance of laboratory animals. The oligonucleotides encoding shRNA targeting FMRP or GFP (Supplemental Table S1) were synthesized and cloned in the lentiviral vector lentilox pLL3.7, as previously described [45]. U251 glioma cells that were uninfected or infected with lentivirus carrying GFP-shRNA or FMRP-shRNA were inoculated subcutaneously into mice (*n* = 13/group). When the xenografts were palpable (about 0.5 cm in diameter), tumor growth was evaluated by monitoring tumor volume (TV=length×width^2^/2) from day 6, every 3 days for 15 days. Relative tumor volumes (RTV) were determined using the relation: RTV=V_i_/V_0_, where V_i_ is the tumor volume measured once every three days and V_0_ is the initial tumor volume measured on the sixth day. RTV was measured at the indicated times. Once the xenograft reached 1.5-3 cm in diameter, the mouse was sacrificed and the xenograft harvested. The harvested xenograft was weighed, and paraffin-embedded sections (4 μm) were prepared for staining with hematoxylin and eosin (H&E) or immunostaining for GFAP, Ki67, and FMRP. The 10 mg tissue of each tumor (5 tumors from each group) was collected and protein expression levels were measured with western blot as described above.

### Statistical analysis

All statistical analyses were performed using SPSS 19.0 for Windows (IBM, Armonk, NY USA). Pairwise comparisons between two groups were made using Student's t-test, while multiple comparisons were made using one-way analysis of variance (ANOVA) followed by the least significant difference (LSD) post-hoc test. Pearson regression analysis was used to correlate FMRP level with Ki67 expression in astrocytoma, whereas the chi-square test was applied to analyze the relationship between the IOD of FMRP^+^ cells and demographics and clinicopathologic data. Kaplan-Meier survival curves were plotted, and statistical comparisons carried out using the log-rank test. All cell culture experiments were performed independently at least three times, and in triplicate each time. A P-value <0.05 was considered statistically significant.

## SUPPLEMENTARY TABLE


