# Supplementary material for: Fragile X mental retardation protein promotes astrocytoma proliferation via the MEK/ERK signaling pathway
Source: Oncotarget. 2016 Sep 23;7(46):75394–406. doi: 10.18632/oncotarget.12215 (PMC5342749; doi:10.18632/oncotarget.12215)
Supplement: Supplementary file 1 [file oncotarget-07-75394-s001.pdf]

Fragile X mental retardation protein promotes astrocytoma proliferation via the MEK/ERK signaling pathway

SUPPLEMENTARY TABLE

Supplementary Table S1: Oligonucleotide sequence of siRNAs.

siRNA	sense	antisense
FMRP siRNA-1	5′-GCAGCCUGAUAGGCAGAUU-3′	5′-AAUCUGCCUAUCAGGCUGC-3′
FMRP siRNA-2	5′-GCAGCUUGCCUCGAGAUUU-3′	5′-AAAUCUCGAGGCAAGCUGC- 3′
GFP siRNA	5′-UAGCGACUAAACACAUCAATT-3′	5′-UUGAUGUGUUUAGUCGCUATT-3′
